# Maternal *Med12* safeguards trophoblast pluripotency and placental development

**DOI:** 10.1093/biolre/ioag066

**Published:** 2026-03-24

**Authors:** Michelle M Halstead, Jyoti Goad, Amina Khan, Oliver Febbo, Rutuja Deshmukh, Aleksandar Rajkovic

**Affiliations:** Department of Pathology, University of California San Francisco, San Francisco, CA, USA; Department of Pathology, University of California San Francisco, San Francisco, CA, USA; Department of Pathology, University of California San Francisco, San Francisco, CA, USA; Department of Pathology, University of California San Francisco, San Francisco, CA, USA; Department of Pathology, University of California San Francisco, San Francisco, CA, USA; Department of Pathology, University of California San Francisco, San Francisco, CA, USA; Department of Obstetrics, Gynecology and Reproductive Sciences, University of California San Francisco, San Francisco, CA, United States; Institute of Human Genetics, University of California San Francisco, San Francisco, CA, USA

**Keywords:** maternal effect gene, mediator, development, placenta, trophoblast, embryonic lethality

## Abstract

For a brief but critical period post-fertilization, the mammalian embryo is entirely dependent on maternal products inherited from the oocyte. Previous research showed that oocyte-specific loss of *Med12*, an X-linked gene and Mediator complex subunit, leads to female sterility despite normal folliculogenesis and ovulation. Here, we show that loss of maternal *Med12* has minimal effect on the oocyte transcriptome and does not manifest in embryonic lethality until post-implantation. Implants derived from *Med12*-null oocytes demonstrate abnormal placentation at E9.5, with an overabundance of trophoblast giant cells (TGC). This phenotype associates with early disruption of lineage markers at the blastocyst stage (e.g., *Pou5f1* and *Gata3*), and later by downregulation of trophoblast pluripotency markers (e.g., *Cdx2*) and activation of drivers of TGC identity (e.g., *Stra13*) in the E7.5 extraembryonic ectoderm, revealing a previously undescribed role for *Med12* in trophoblast pluripotency maintenance. Notably, we find consistently low *Med12* expression in embryos derived from *Med12*-null oocytes, likely due to programmed paternal X chromosome inactivation (XCI). To isolate the consequences of maternal *Med12* depletion, we introduced an autosomal *Med12* transgene and show that embryonic expression of the transgene rescues development of *Med12*-null oocytes. We conclude that oocyte-specific deletion of *Med12* produces a maternal-zygotic double knock-out in extraembryonic tissues due to paternal XCI, leading to loss of pluripotency in the trophoblast, placental malformation, and embryonic death.

## Graphical Abstract

**Graphical Abstract:**
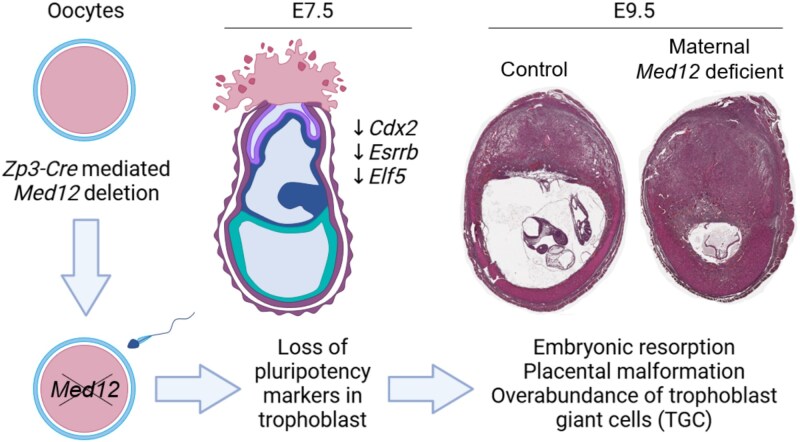

## Teaser

Oocyte-specific *Med12* knockout disrupts trophoblast development, leading to an overabundance of trophoblast giant cells, abnormal placentation, and embryonic lethality.

## Introduction

Early pregnancy loss (EPL) remains a significant challenge in human reproductive medicine, affecting about 10% of clinically recognized pregnancies [1]. An estimated 80% of miscarriages occur in the first trimester of pregnancy [2], often before the person knows they are pregnant. However, very little is known about the mechanisms that govern successful development during early embryogenesis. Maternal effect genes (MEG), which encode oocyte-derived factors that are dispensable for oogenesis but essential for embryogenesis, are of particular interest, as EPL driven by MEG dysregulation is determined solely by the mother’s genotype. Maternal effect genes guide embryonic development before the onset of embryonic transcription, regulating crucial processes such as zygotic genome activation (ZGA), epigenetic reprogramming, and cell differentiation [3]. Disruption of MEG can profoundly impact embryonic development, potentially leading to EPL or birth defects. However, relatively few MEG have been described in mammals.

Using Zp3Cre-mediated recombination, we previously demonstrated that oocyte-specific depletion of Mediator complex subunit 12 (*Med12*) leads to female sterility in mice without impacting folliculogenesis or ovulation [4]. This finding suggests that *Med12* may play a novel role as an MEG in mice, but it remains unclear when and how the loss of oocyte-derived *Med12* causes female sterility. The Mediator complex is a critical determinant of overall genome organization and gene expression, acting as a bridge between gene-specific transcription factors bound to enhancer elements and the RNA polymerase II transcription machinery at gene promoters [5, 6]. Structurally, the Mediator complex is organized into three core modules—head, middle, and tail—comprising 26 subunits, along with a detachable kinase module consisting of MED12, MED13, Cyclin C, and CDK8, which regulates the function of the core Mediator [7–10]. MED12 is essential for activation of the kinase module, which can modulate the interaction of Mediator with RNA polymerase II, thereby influencing transcription [11–15] and chromatin architecture [5, 6, 16–19] in a context-dependent manner. Tetraploid complementation experiments showed that *Med12*-null embryonic stem cells (ESCs) fail to support development, with defects in Wnt signaling and gastrulation leading to embryonic lethality [20]. However, the role of maternal *Med12* in the preimplantation embryo and extraembryonic tissues has not been investigated. Given *Med12*’s essential role in transcriptional regulation, its loss in oocytes may disrupt early developmental processes, such as ZGA and differentiation of the trophectoderm and inner cell mass.

To investigate the role of maternal *Med12* in the oocyte and early embryo, we ablated *Med12* from primary follicles via Zp3Cre-mediated recombination and investigated the molecular signatures of the resulting oocytes and embryos. Loss of maternal *Med12* led to placental malformation and downregulation of pluripotency markers in the extraembryonic ectoderm, implicating *Med12* in trophoblast pluripotency maintenance. Notably, embryonic expression of an autosomal *Med12* transgene rescued development of *Med12*-null oocytes, suggesting that the requirement for oocyte-derived *Med12* was rooted in the rodent-specific phenomenon of paternal X chromosome inactivation (XCI).

## Materials and methods

### Experimental mice

All procedures were approved by the IACUC of the University of California, San Francisco (UCSF) and conducted in accordance with NIH and UCSF guidelines for the care and use of laboratory animals (IACUC protocol AN204752-00C). Mice were housed under standard conditions with a 12 h light–dark cycle and provided ad libitum access to food and water. *Med12^flox^* mice were a gift from Dr. Heinrich Schrewe (Max–Planck Institute, Berlin, Germany) [20], and were maintained on a C57BL/6J/129SV background. *Zp3Cre* mice were purchased from Jackson Laboratories (Stock #003651) and were maintained on a C57BL/6J background [21]. *Med12* autosomal knock-in mice were generated as previously described by subcloning full-length *Med12* cDNA into the pROSA26-DV1 vector and inserting into the autosomal *Rosa26* locus [22]. *Med12* knock-in mice were maintained on an FVB/C57BL/6/129SV background. To test dam fertility, single females of 6- to 10-weeks of age were continuously housed with stud males of proven fertility for up to 6 months, during which time the number of litters, litter size, and sex ratios of pups were recorded. Genotyping was performed following standard PCR protocols and using DNA extracted from either tail or ear biopsies (Supplementary Table S2).

### Histological analysis and immunohistochemical staining

Uteri harvested at various stages of gestation were fixed in 10% formalin overnight, embedded in paraffin, and serially sectioned at 5 μm thickness using a HistoCore MULTICUT microtome (Leica Biosystems). Sections were stained with hematoxylin and eosin for histological analysis. For immunohistochemistry, paraffin sections were heated for 30 min at 55°C, dewaxed with xylene, rehydrated, and washed. For antigen retrieval, slides were then incubated in Tris-EDTA buffer (10 mM Tris, 1 mM EDTA, 0.05% Tween, pH 9.0) in a decloaking chamber at 125°C for 4 min, then 90°C for 10 s, incubated at room temperature for 20 min, and washed with distilled water for 5 min. Slides were then quenched in 3% H_2_O_2_ in methanol for 20 min at room temperature, and washed 2x 5 min in 1X PBS-T. Slides were blocked in 10% goat serum (Sigma–Aldrich, G9023) in 1X PBS-T for 1 h at room temperature and then incubated with primary antibody diluted in blocking buffer overnight at 4°C: anti-CDX2 (Abcam, ab76541) at a 1/1000 dilution; anti-TPBPA (Abcam, ab320823) at a 1/1000 dilution; anti-CEBPA (Abcam, ab317442) at a 1/2000 dilution. Negative controls were performed by omitting the primary antibody to assess non-specific background staining. The next day, slides were washed 3x 5 min in 1X PBS-T, then incubated with Biotin-SP conjugated goat anti-rabbit (Jackson ImmunoResearch Laboratories, 111-065-003) at a 1/250 dilution in blocking buffer for 1 h at room temperature. Slides were washed 3x 5 min in 1X PBS-T, then incubated with peroxidase conjugated streptavidin (Jackson ImmunoResearch Laboratories, 016-030-084) at a 1/250 dilution in 1X PBS-T for 1 h at room temperature. Slides were washed 3x 5 min in 1X PBS-T, developed with the NovaRED Substrate Kit (Vector Laboratories, SK-4800) for 5 min, then counterstained with modified Mayer hematoxylin (Fisher Scientific, 22-110-639), rehydrated, and mounted. Images were captured with the Aperio GT 450 scanner system (Leica Biosystems) and processed with Aperio ImageScope software (v.12.4.6.5003).

### Oocyte collection for RNA-seq

Ovaries from 12 day old females were incubated in digestion media (1X DPBS, 2 mg/mL collagenase type 1 (Sigma, SCR103), 2 mg/mL BSA (Sigma, A3311)) at 37°C for 1 h, then supplemented with 40 mM EDTA (pH 8.0) and incubated at 37°C for 10 min. Digested ovaries were centrifuged at 1000 rpm for 5 min, and cell pellets resuspended in pick-up media (1x MEM-Alpha (Gibco, 12561056), 25 mM NaHCO_3_, 2 mg/mL BSA). Oocytes were transferred to wash buffer (1x MEM-Alpha, 25 mM NaHCO_3_, 1 mg/mL BSA), then to 1x DPBS, and stored in media at −80°C. For GV and MII oocyte collection, female mice older than 6 weeks were synchronized by 5 IU PMSG (BioVendor, RP178272100). For GV oocytes, ovaries were collected 48 h after PMSG administration and transferred to collection media (1X Hepes-MEM (Gibco, 12360038), 6 mM NaHCO_3_, 0.23 mM sodium pyruvate, 3 mg/mL BSA) with 1 μM cilostamide (Sigma, 231085). Follicles were mechanically disrupted using two needles and oocytes were selected based on size and morphology. Oocytes were washed in maturation media (1X MEM-Alpha, 6 mM NaHCO_3_, 3 mg/mL BSA) with 1 μM cilostamide, then in 1X DPBS, and stored in Trizol solution (ThermoFisher, 15596026) at −80°C. For MII oocytes, female mice were injected with 5 IU hCG (Sigma, C1063) 48 h after PMSG, and oocytes were collected 12–15 h after hCG. Ovaries were transferred to collection media. After follicle disruption, cumulus complexes were transferred to maturation media and denuded with 0.3 mg/mL hyaluronidase (Sigma, H3506) for 1 min. Denuded oocytes were washed in maturation media, then 1X DBPS, and stored in Trizol at −80°C.

### Embryo collection for RNA-seq

Female mice older than 6 weeks were synchronized by 5 IU PMSG for 48 h, then injected with 5 IU hCG and mated with wild-type stud males of proven fertility. For blastocyst collection, reproductive tracts were collected from euthanized female 4 days post hCG and transferred to 1X DPBS supplemented with 0.1% BSA. Individual blastocysts were flushed from oviducts, washed to remove debris, and stored in 1X Lysis Buffer (Takara Bio) supplemented with RNase Inhibitor (Takara Bio) [per embryo, 0.95 μL 10X Lysis Buffer, 10.5 μL nuclease-free water, 0.05 μL RNase Inhibitor] at −80°C prior to library preparation. For E7.5 implant collection, uteri were collected from euthanized females 8 days post hCG and transferred to HEPES-buffered DMEM supplemented with 10% FBS (Cytiva, SH30088.03). Each embryo was carefully separated from the maternal decidua and dissected into the ectoplacental cone, extraembryonic ectoderm, and epiblast. Tissues were stored in Trizol solution at −80°C prior to RNA extraction.

### RNA-seq library preparation and sequencing

For oocytes and E7.5 tissue, total RNA was extracted from samples stored in Trizol solution by phenol-chloroform extraction. The aqueous phase was subjected to column purification using the PicoPure RNA Isolation kit (ThermoFisher, KIT0204) and treated with DNase I (Qiagen, 79254) to remove genomic DNA. For samples not stored in Trizol, total RNA was extracted using the Extraction Buffer provided in the PicoPure RNA Isolation kit. RNA integrity (RQN > 7) was evaluated with the 5200 Fragment Analyzer System (Agilent Technologies). For oocyte samples, libraries were constructed with the SMART-Seq v4 Ultra Low Input RNA kit (Takara Bio) and sequenced on the HiSeq 4000 platform to generate 50 bp single-end reads. For blastocysts, libraries were constructed from individual embryos with the SMART-Seq v4 Ultra Low Input RNA Kit (Takara Bio) and sequenced on the NovaSeq X platform to generate 50 bp paired-end reads. For E7.5 embryo samples, libraries were constructed with the Tecan Universal Plus Total RNA library preparation kit (Tecan Life Sciences) and sequenced on the NovaSeq X platform to generate 50 bp single end reads.

### RNA-seq data analysis

Raw reads were trimmed with Trim_Galore (v0.6.7) and aligned to the GRCm39 assembly with STAR (v2.7.10b). Low quality alignments (q < 5) were removed with SAMtools (v1.16) and raw counts for genes in the Ensembl (v108) annotation were calculated with HTSeq (v2.0.2) in mode “intersection-nonempty”. Raw counts were log transformed with the R package DESeq2 (v1.36.0). Principal components analyses were conducted with the plotPCA function, considering the top 5000 genes with highest variable expression. Pearson correlations were calculated with cor function from the R package stats (v4.3.3) and plotted with pheatmap (v1.0.12). Differentially expressed genes were identified with DESeq2 using an adjusted *P* value threshold of 0.01. For oocytes, pairwise comparisons were conducted to compare samples from mutant and control dams for each stage (D12, GV, MII). For blastocysts, embryos from mutant dams were compared to those from control dams (including all samples) and in a pairwise fashion by embryonic sex (male or female). For E7.5 embryos, separate pairwise comparisons were conducted to compare samples from mutant and control dams for each embryonic tissue (EPC, ExE, EPI) and embryonic sex (male or female). Volcano plots were generated with EnhancedVolcano (v1.20.0). Functional enrichment of gene sets was calculated using the DAVID webtool (v2024q1). Bar plots and bubble plots were generated with ggplot2 (v3.5.1) and ggpubr (v0.6.0).

### Oocyte collection for quantitative PCR

Female mice older than 6 weeks were synchronized by 5 IU PMSG. For GV oocytes, ovaries were collected 48 h after PMSG administration and transferred to warmed M2 media (Sigma, M7167). Follicles were mechanically disrupted using two needles and oocytes were selected based on size and morphology. For MII oocytes, mice were injected with 5 IU hCG 48 h after PMSG, and oocytes were collected 12–15 h after hCG. Cumulus oocyte complexes (COCs) were isolated from the ampulla in warmed M2 media. COCs were denuded with 0.3 mg/mL hyaluronidase for 1 min. After isolation, oocytes were washed 3x in M2 media, frozen in minimal media (<5 μL) on dry ice, then stored at −80°C. Oocytes were lysed using the SingleShot Cell Lysis kit (BioRad, 172-5080). To each sample, 10 μL of lysis mix (9.6 μL SingleShot Cell Lysis buffer, 0.2 μL proteinase K, 0.2 μL DNase I) was added, and samples were incubated 15 min at 22°C, 5 min at 37°C, then 5 min at 75°C. Lysates were immediately subjected to reverse transcription with the SuperScript III First-Strand Synthesis System using random primers (ThermoFisher, 18080-051).

**Figure 1:**
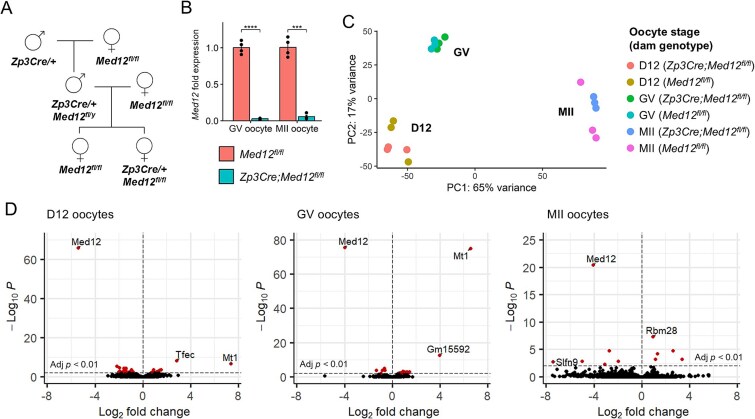
*Med12* ablation does not affect the oocyte transcriptome. (A) Breeding scheme to obtain control (*Med12^fl/fl^*) and mutant (*Zp3Cre;Med12^fl/fl^*) dams. (B) Relative abundance ± S.E.M. of *Med12* in GV and MII oocytes by RT-qPCR. Calculated by the ΔΔCt method using the reference gene *Gapdh* (*n* = 4 replicates per genotype/stage)*.* One-tailed unpaired Student t-test (^***^P < 0.001, ^****^P < 1e-4). (C) Principal components analysis of growing (day 12; D12), GV and MII oocyte transcriptomes from *Med12^fl/fl^* and *Zp3Cre;Med12^fl/fl^* dams. Based on top 5000 genes with highest variable expression. (D) Differential gene expression in oocytes from control versus mutant dams. Differentially expressed genes colored red (adjusted *P* < 0.01).

### Tissue processing from adult mice

Tissues from adult male mice were harvested at 10 weeks of age. Brain, heart, liver, and testis were weighed and stored in Trizol solution at −80°C. Total RNA was extracted by phenol-chloroform and purified with the RNeasy Mini kit (Qiagen, 74104) and DNase I (Qiagen, 79254). RNA extraction from brain was modified as previously described [23]. Briefly, after chloroform extraction an equal volume of isopropylalcohol was added to the aqueous phase, and samples were centrifuged to pellet total RNA, which was resuspended in 70% ethanol before purification with the Qiagen RNeasy Mini kit. Purified RNA was subjected to reverse transcription with the SuperScript III First-Strand Synthesis System using random primers (ThermoFisher, 18080-051).

### Real-time quantitative PCR

Quantitative PCR was performed on the StepOnePlus instrument (ThermoFisher) using the SsoAdvanced Universal SYBR Green Supermix (Biorad, 1,725,271) (Supplementary Table S3). Samples were run in quadruplicate. C_T_ values were normalized to *Gapdh* and relative transcript abundance was determined with the ΔΔC_T_ method. Briefly, for each sample, the ΔCt value was calculated as the difference between the average Ct of the target gene and the average Ct of *Gapdh* (ΔCt = Ct_target – Ct_Gapdh). Then, the ΔΔCt value was obtained by subtracting the average ΔCt of the control group from the ΔCt of each sample (ΔΔCt = ΔCt_sample – ΔCt_control). Fold changes in gene expression were calculated as 2^–ΔΔCt^.

### Statistics

Student *t* tests were conducted with the t_test function from the rstatix package (v0.7.2). *P* values were adjusted for multiple testing using the Holm-Bonferroni method. An adjusted *P* value less than 0.05 was considered statistically significant.

## Results

### Med12 does not regulate transcription in the oocyte

Considering the fundamental role of *Med12* in transcriptional regulation, we hypothesized that oocyte-specific knock-out of *Med12* would alter the oocyte transcriptome and thereby compromise embryonic development. To address this hypothesis, *Med12* was ablated from primary follicles via Zp3Cre-mediated recombination as reported previously [4, 24] (Figure 1A). As expected, real-time quantitative PCR (RT-qPCR) showed that *Med12* transcripts were significantly reduced by about 20-fold in germinal vesicle (GV) and metaphase II (MII) stage oocytes from *Zp3Cre;Med12^fl/fl^* dams compared to *Med12^fl/fl^* control dams (Figure 1B). To determine the consequences of *Med12* depletion on transcription regulation in the oocyte, oocytes from 12 day old (D12) and adult females (GV and MII oocytes) were collected for bulk RNA-seq (Figure 1C, Supplementary Figure S1). Despite the core role of the Mediator complex in transcription regulation, *Med12* deficiency had very little effect on the oocyte transcriptome. Only 33, 19, and 10 genes were dysregulated (adjusted *P* < 0.01) in D12, GV, and MII stage *Med12*-deficient oocytes, respectively (Figure 1D, Supplementary Data S1). Notably, *Mt1* was upregulated in both D12 and GV mutant oocytes; however, upregulation of *Mt1* may indirectly result from Zp3Cre transgene expression [24], rather than downregulation of *Med12*. Otherwise, a select few genes were notably upregulated in *Med12*-null oocytes: the transcription factor *Tfec* in D12 oocytes, the lncRNA *Gm15592* in GV oocytes, and an RNA-binding protein *Rbm28* in MII oocytes. However, the upregulation of these specific factors was not consistent across stages and did not incur larger-scale disruptions of the oocyte transcriptome prior to fertilization. Overall, *Med12* does not appear to play an important role in transcriptional regulation in the oocyte.

**Figure 2:**
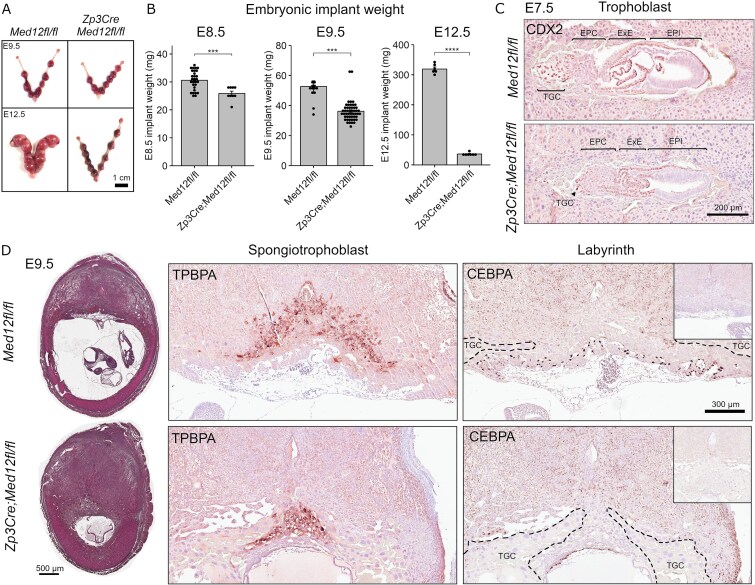
Maternal *Med12* is required for trophoblast development. Uteri collected between E7.5 and E12.5 from control (*Med12^fl/fl^*) and mutant (*Zp3Cre;Med12^fl/fl^*) females mated with wild-type males. (A) Gross analysis of embryo implants at E9.5 and E12.5, showing hemorrhagic sites and embryo reabsorption in mutant dams at E12.5. (B) Weight of individual embryo implants ± S.E.M. at E8.5 (*n* = 29 from control dams; *n* = 9 from mutant dams), E9.5 (*n* = 13 from control dams; *n* = 50 from mutant dams), and E12.5 (*n* = 6 from control dams; *n* = 7 from mutant dams). Two-tailed unpaired Student t-test (^***^P < 0.001, ^****^P < 1e-4). (C) Immunohistochemical staining of E7.5 embryo implants from control and mutant dams for CDX2 (nuclear marker of trophoblast pluripotency). EPC, ectoplacental cone; ExE, extraembryonic ectoderm; EPI, epiblast; TGC, trophoblast giant cells. (D) Histological analysis and immunohistochemical staining of E9.5 embryonic implants from control and mutant dams. Implants stained for TPBPA (cytoplasmic marker of spongiotrophoblast layer) and CEBPA (nuclear marker of labyrinthine trophoblast). Implantation sites from Zp3Cre; Med12^fl/fl^ dams exhibited an overabundance of TGC and reduced spongiotrophoblast and labyrinth layers. Arrowheads indicate the reduced labyrinth layer in mutant dams, consisting of compacted CEBPA-positive cells. Negative controls (no primary antibody) are shown in subpanels.

### Maternal Med12 is required for trophoblast development

Transcription was not dysregulated in *Med12*-deficient oocytes, suggesting that maternal *Med12* is not required until after fertilization. To understand when and how loss of maternal *Med12* leads to female sterility, control (*Med12^fl/fl^*) and mutant (*Zp3Cre;Med12^fl/fl^*) dams were timed-mated with wild-type stud males and uteri were collected between E7.5 and E12.5. Similar numbers of implantation sites were observed in control and mutant dams (Supplementary Figure S2, Supplementary Table S1). At E9.5, there were no gross morphological differences in implants between control and mutant dams; however, by E12.5 hemorrhagic sites and embryo reabsorption were observed in mutant dams (Figure 2A). As early as E8.5, implants were significantly smaller in mutant dams, and by E12.5, control implants were 10 times larger than mutant implants (Figure 2B). At E7.5, implants from control and mutant dams had similar gross morphology with the expected compartments: epiblast (EPI), extraembryonic ectoderm (ExE), and ectoplacental cone (EPC) (Figure 2C). However, E7.5 embryos from mutant dams lacked the typical cluster of secondary TGC distal to the EPC (Figure 2C). Phenotypic abnormalities were more evident at E9.5, at which point mutant implants already showed evidence of embryonic demise (Figure 2D). Moreover, the placental morphology of mutant implants was irregular, with excessive numbers of TGC, diminished spongiotrophoblast, and compacted labyrinth layers (Figure 2D). Given the key roles of these tissues in supporting embryonic growth, this abnormal placental development could explain the reduced implant weight and eventual embryonic resorption by E12.5.

**Figure 3:**
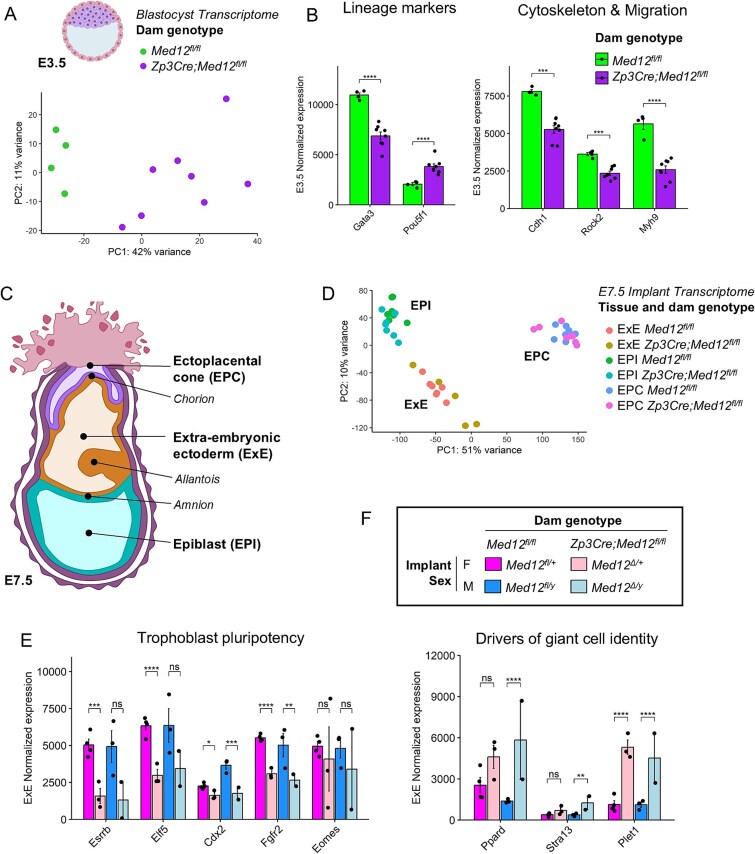
RNA-seq of E7.5 embryos lacking maternal *Med12*. (A) Principal components analysis of E3.5 blastocyst transcriptomes, based on top 5000 genes with highest variable expression. Samples are colored based on dam genotype. (B) Normalized expression ± S.E.M. of lineage markers and genes involved in cytoskeletal organization and migration in blastocysts. (C) Representation of an E7.5 embryonic implant. (D) Principal components analysis of E7.5 transcriptomes, based on top 5000 genes with highest variable expression. Samples are colored based on E7.5 tissue and dam genotype. (E, F) Normalized expression ± S.E.M. of trophoblast pluripotency markers (E) and giant cell markers (F) in E7.5 ExE. Adjusted *P*-values from pairwise differential expression analyses (ns *P* ≥ 0.05; ^*^P < 0.05; ^**^P < 0.01; ^***^P < 0.001; ^****^P < 1e-4).

The morphological abnormalities observed in *Med12*-deficient implants could reflect disruptions to transcription regulation in the lineages that give rise to TGC and other placental cell types. To understand how the loss of maternal *Med12* disrupts placental development at a molecular level, control (*Med12^fl/fl^*) and mutant (*Zp3Cre;Med12^fl/fl^*) dams were timed-mated with wild-type stud males and individual E3.5 blastocysts with normal morphology and an expanded blastocoel were collected for bulk RNA-seq. Based on gene expression, embryos were sexed and *Med12* knock-down was confirmed (Supplementary Figure S3). Blastocyst transcriptomes segregated by maternal genotype (Figure 3A) and differential expression analysis of blastocysts from control (*n* = 4) and mutant dams (*n* = 7) revealed 1221 dysregulated genes (adjusted *P* < 0.01) (Supplementary Data S2). Upregulated genes in *Med12*-deficient embryos were involved in transcriptional regulation and chromatin-associated processes, and downregulated genes had functions tied to cytoskeletal organization and epithelial morphogenesis (Figure 3B, Supplementary Data S3). Notably, the transcription factor *Pou5f1*, which is crucial for maintenance of epiblast pluripotency, was upregulated in *Med12*-deficient blastocysts, whereas *Gata3*, which promotes trophoblast lineage identity, was downregulated (Figure 3B). Moreover, the trophectoderm-specific receptor *Fgfr2*, which mediates FGF4 signaling from the epiblast and is crucial for maintaining the primitive endoderm and trophectoderm, was significantly downregulated in the trophectoderm of *Med12*-deficient embryos (Supplementary Figure S4A). In general, epiblast-specific markers tended to be upregulated in *Med12*-deficient embryos, and trophectoderm-specific markers were downregulated (Supplementary Figure S4). This imbalance suggests that loss of maternal *Med12* causes a failure to silence epiblast programs in the trophectoderm and a dampening of trophoblast-specific programs at the blastocyst stage.

Next, we examined consequences of maternal *Med12* loss to the E7.5 transcriptome. Individual E7.5 implants from control and mutant dams were dissected into the EPI, ExE, and EPC for RNA-seq (Figure 3C). Based on gene expression, embryos were sexed and *Med12* knock-down was confirmed (Supplementary Figure S5). Overall, E7.5 samples clearly clustered by embryonic tissue of origin but did not clearly segregate by maternal genotype (Figure 3D). Pairwise comparisons between embryos lacking maternal *Med12* and controls were conducted for each embryonic tissue and sex to identify differentially expressed genes (Table 1, Supplementary Figure S6, Supplementary Data S4). Overall, loss of maternal *Med12* led to dysregulation of core cellular processes, including metabolism, translation, and transcription (Supplementary Figure S7). Consistent with previous reports of *Med12* knock-out embryos generated through tetraploid complementation [20], depletion of *Med12* in the epiblast disrupted Wnt signaling and somitogenesis. Echoing the transcription dysregulation at the blastocyst stage, genes involved in cell differentiation, proliferation, migration, and cytoskeletal organization were broadly dysregulated in all E7.5 tissues of *Med12*-depleted embryos (Supplementary Figures S7, S8). The ExE, which gives rise to specialized placental cell types, was particularly impacted. Markers of trophoblast pluripotency (*Cdx2, Esrrb, Fgfr2, Eomes, Elf5*) were downregulated in the ExE of mutant embryos (Figure 3E), whereas drivers of giant cell identity (*Stra13, Ppard, Plet1*) were upregulated (Figure 3F). To assess whether maternal *Med12* loss alters the balance between mural and polar trophectoderm (TE) identity, we examined expression of *Tfap2a* (expressed in both mural and polar TE) and *Tfap2c* (specific to mural TE) in E7.5 extraembryonic tissues. The balance between mural and polar TE is important because mural TE gives rise to TGC, whereas polar TE contributes to the ExE and labyrinth. Although differences were not statistically significant, *Tfap2a* expression was modestly reduced and *Tfap2c* somewhat increased in the ExE of *Med12*-deficient embryos (Supplementary Figure S8). These trends suggest that maternal *Med12* loss may impair pluripotency maintenance and promote a shift toward mural-like identity in the ExE, potentially predisposing cells to premature TGC differentiation. Overall, the transcriptomic perturbations in E7.5 extraembryonic tissues were consistent with the overabundance of TGC observed at E9.5 (Figure 2D). Placental dysmorphogenesis of embryonic implants in *Zp3Cre;Med12^fl/fl^* mutant dams therefore appears to be linked to compromised trophoblast pluripotency maintenance and misspecification of trophoblast subtypes.

**Table 1:** Differentially expressed genes (DEG) between E7.5 embryos from control (*Med12^fl/fl^*) and mutant (*Zp3Cre;Med12^fl/fl^*) dams (adj *P* < 0.01).

E7.5 tissue	Ectoplacental cone			Extraembryonic ectoderm			Epiblast		
Embryo sex in which DEG identified	Female	Male	Both	Female	Male	Both	Female	Male	Both
Upregulated DEG in embryos depleted for maternal *Med12*	165	252	67	794	616	350	496	504	248
Downregulated DEG in embryos depleted for maternal *Med12*	142	232	56	740	677	289	182	485	94

Pairwise comparisons conducted for each embryonic tissue and sex. DEG grouped based on whether they were identified only when comparing males, only females, or in both sexes.

### Embryonic expression of a Med12 transgene compensates for loss of maternal Med12

Our previous research showed that *Zp3Cre;Med12^fl/fl^* females are sterile when mated with wild-type stud males [4]. Because *Med12* is X-linked and likely subject to programmed paternal XCI in mice [25], we hypothesized that embryos from *Zp3Cre;Med12^fl/fl^* females lack *Med12* expression in extraembryonic tissues, producing a functional maternal-zygotic knockout. This interpretation is supported by the consistently low levels of *Med12* in male (*Med12^Δ/y^*) and female (*Med12^Δ/+^*) embryos from mutant dams, suggesting that transcription from the paternal X chromosome in female embryos was insufficient to restore *Med12* expression to normal levels (Supplementary Figures S3 and S5). In this case, oocyte-specific knock-out cannot evaluate the specific role of maternal *Med12* without also depleting embryonic *Med12*, and an alternative strategy was required to restore embryonic *Med12* expression while simultaneously depleting maternal stores.

**Figure 4:**
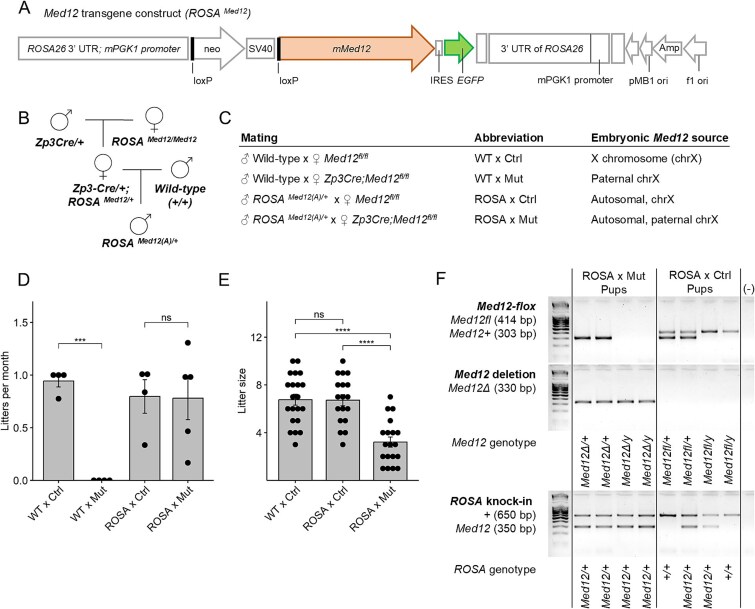
Embryonic expression of a *Med12* transgene compensates for loss of maternal *Med12*. (A) *Med12* transgene construct for knock-in to the *Rosa26* safe harbor locus on chromosome 6. (B) Breeding strategy to obtain stud males that express the activated *Med12* transgene (e.g., *ROSA^Med12(A)^*). (C) Mating strategies to test fertility of control (*Med12^fl/fl^*) and mutant (*Zp3Cre;Med12^fl/fl^*) dams, and corresponding source(s) of *Med12* in resultant embryos. (D) Litters produced per month ± S.E.M. for each breeding pair and (E) litter size ± S.E.M. for each mating combination. One-tailed unpaired Student t-test, adjusted by Holm–Bonferroni method (ns *P* ≥ 0.05; ^*^P < 0.05; ^**^P < 0.01; ^***^P < 0.001; ^****^P < 1e-4). (F) Representative genotyping of pups produced from matings between males with an active *Med12* transgene and control or mutant dams.

To address the complication of paternal XCI, we generated transgenic mice that express *Med12* from an autosome, as described previously [22]. Briefly, the *Med12* construct was knocked into the *Rosa26* safe harbor locus on chromosome 6. The *Med12* transgene (*ROSA^Med12^*), driven by the murine *PGK1* promoter, was designed such that it could be transcriptionally activated (*ROSA^Med12(A)^*) through Cre-mediated recombination (Figure 4A).

Using this strategy, we obtained stud males that express *Med12* from an autosome (Figure 4B) and mated them with mutant (*Zp3Cre;Med12^fl/fl^*) and control (*Med12^fl/fl^*) dams to test whether embryonic expression of the paternal autosomal *Med12* transgene could compensate for the loss of maternal *Med12* (Figure 4C)*.* As previously observed, mutant dams were infertile when mated with wild-type males; however, when mated with males that expressed the *Med12* transgene (*ROSA^Med12(A)/+^*), mutant dams produced litters at a similar frequency to control dams (Figure 4D), although litter size was significantly smaller (Figure 4E). Every pup born to a (*Zp3Cre;Med12^fl/fl^*) dam and (*ROSA^Med12(A)/+^*) sire carried a *ROSA^Med12(A)^* allele and an endogenous *Med12* deletion (*n* = 29/29 pups), whereas only half of pups born to (*Med12^fl/fl^*) dams inherited the *ROSA^Med12(A)^* allele (*n* = 16/29 pups), and all carried the intact *Med12^flox^* allele (Figure 4F). This observation explains why the average litter size for mutant dams (3.67 pups) was about half of that observed in control dams (6.17 pups). Based on Mendelian ratios, 50% of embryos in mutant dams would not inherit the *ROSA^Med12(A)^* allele from the sire and would perish before E12.5.

Surprisingly, pups born to (*Zp3Cre;Med12^fl/fl^*) dams and (*ROSA^Med12(A)/+^*) sires were both viable and fertile (Supplementary Figure S9A and B), with no apparent phenotypic consequences resulting from *Med12* transgene expression (Supplementary Figure S9C). Moreover, *Med12* expression in (*ROSA^Med12(A)/+^*) stud males and their male offspring (*Med12^Δ/y^;ROSA^Med12(A)/+^*) was significantly higher than in wild-type counterparts (Supplementary Figure S9D), suggesting that mice are highly robust to *Med12* overexpression and/or that post-transcriptional mechanisms exist to control MED12 protein levels. Overall, embryonic expression of the autosomal *Med12* transgene was sufficient and necessary to rescue development of embryos lacking endogenous maternal *Med12*. This finding suggests that the sterility of *Zp3Cre;Med12^fl/fl^* dams results from the combined loss of maternal *Med12* and persistent inactivation of the paternal *Med12* allele in the preimplantation embryo and extraembryonic tissues. The rescue of development by the paternal autosomal *Med12* transgene further indicates that *Med12* is not required in the oocyte itself, but rather that maternal *Med12* is required post-fertilization to support early embryo development.

## Discussion

This study identifies *Med12* as an MEG required for preimplantation embryo development and proper placentation. Through oocyte-specific *Med12* knock out, we find that loss of maternal *Med12* leads to early transcriptional imbalances at the blastocyst stage, with upregulation of epiblast markers (e.g., *Pou5f1*) and downregulation of trophectoderm markers (e.g., *Gata3*). These changes precede a collapse of trophoblast pluripotency in the ExE by E7.5, characterized by loss of pluripotency regulators and upregulation of TGC differentiation markers. By E9.5, the placenta shows abnormal architecture: excessive TGCs, diminished spongiotrophoblast, and compressed labyrinth. These placental defects ultimately result in embryonic demise and resorption between E9.5 and E12.5. Notably, this phenotype is due to loss of both maternal and embryonic *Med12*, likely driven by programmed paternal XCI. Together, our data reveal a critical role for *Med12* in stabilizing transcriptional and signaling programs that preserve lineage boundaries and support placental development.

Most MEG act during oogenesis, fertilization, or cleavage stages to enable key crucial processes such as ZGA and the first embryonic divisions [3]. In contrast, *Med12*-null oocytes support fertilization and preimplantation development. Very few genes are dysregulated in *Med12*-null oocytes and it appears that *Med12* is not essential for initiating embryonic transcription but instead plays a later role in lineage specification. *Med12* therefore joins a small group of later-acting MEG, including *Ezh1* and *Dnmt3l*, which affect blastocyst-stage development and beyond, often through epigenetic mechanisms [26, 27]. Given known interactions between MED12 and chromatin modifiers such as histone acetyltransferases [28], the loss of maternal *Med12* may disrupt enhancer activity and chromatin accessibility in the preimplantation embryo, thereby disrupting gene expression programs that are necessary later in development.

Indeed, MED12 has been implicated in enhancer regulation across several stem cell contexts. In ESCs, it scaffolds enhancer-promoter loops through the Mediator-cohesin complex to sustain pluripotency gene expression [5, 6, 18]. In hematopoietic stem cells, MED12 preserves stemness by maintaining histone acetylation at lineage-specific enhancers [19]. In trophoblast stem cells (TSCs), considered analogous to ExE [29, 30], MED12 co-localizes with acetyltransferases at super-enhancers [31], suggesting a similar regulatory role in the trophoblast lineage, although the functional consequences of MED12 loss in TSCs remain untested. Our findings reveal that maternal *Med12* is similarly required to maintain stem-like transcriptional programs in vivo, suggesting enhancer dysregulation as a unifying mechanism.

However, the effects of *Med12* loss varied across lineages: in the E7.5 ExE, pluripotency genes were downregulated, whereas in the E7.5 epiblast, Wnt signaling was disrupted, indicating that MED12 activity is shaped by cell-type-specific regulatory environments. Future studies using epigenomic profiling in early embryos will be essential to determine whether enhancer activity is directly impacted by maternal Med12 loss, and how that varies between lineages. Such specificity may arise from cooperation with lineage-specific transcription factors like CDX2 or ELF5, recruitment to cell-specific enhancers, or functions independent of the canonical Mediator complex. In other systems, MED12 interacts directly with other transcription factors to regulate gene expression [32, 33], suggesting that similar partnerships may enable its context-dependent roles in sustaining ExE identity and preventing premature differentiation.

Notably, the transcriptional consequences of Med12 depletion also diverge across in vivo and in vitro contexts. In ESCs, *Med12* knockdown leads to reduced *Pou5f1* expression [18], whereas we found that maternal *Med12* deficiency in embryos causes *Pou5f1* upregulation in blastocysts. This discrepancy may reflect differences in regulatory network topology or the absence of intercellular signaling in ESC monoculture. For example, in *Med12*-deficient embryos, upregulation of *Pou5f1* could be driven by ectopic expression in the trophectoderm, potentially due to downregulation of key trophoblast factors that maintain lineage boundaries in the blastocyst [34, 35]. These changes suggest that *Med12* loss disrupts not only cell-intrinsic enhancer regulation but also broader signaling environments. Disruption of cell–cell signaling is also exemplified by defects in FGF4 signaling: in *Med12*-deficient E3.5 blastocysts, *Fgfr2* is significantly downregulated, and by E7.5, *Fgf4* expression is reduced in the epiblast while *Fgfr2* remains low in the ExE. As FGF4 from the epiblast is essential for maintaining trophoblast stemness via *Cdx2* induction [36], disruption of FGF4 signaling likely contributes to ExE collapse and premature differentiation at E9.5. Together, these findings suggest that maternal *Med12* supports pluripotency and lineage boundaries through both enhancer activity and cell–cell communication.

Although we detected transcriptomic perturbations in Med12-deficient blastocysts and E7.5 implants, there were no obvious phenotypic abnormalities until E9.5. This delay may reflect the time required for the proteome to catch up with transcript-level changes. Such an explanation aligns well with the dysregulation of pluripotency and TGC marker genes observed in E7.5 ExE and the subsequent bias towards TGC identity in E9.5 placenta. In TSCs, loss of any one of the pluripotency genes downregulated in E7.5 ExE (*Esrrb, Cdx2, Elf5*, and *Eomes* [37–39]) induces differentiation toward the TGC fate [40, 41], and ectopic expression of any of the TGC markers that were upregulated (*Stra13, Ppard*, and *Plet1* [42–45]) induces terminal differentiation into giant cells [42–45]. Moreover, *Plet1* promotes TGC identity while suppressing syncytiotrophoblast fate [45]. This molecular cascade likely underlies the placental defects we observed at E9.5, including TGC overabundance and diminished syncytiotrophoblast and labyrinth layers. These latter compartments are essential for hormone secretion, maternal-fetal exchange, and nutrient transport [46], and their depletion likely compromises placental function, leading to fetal demise. Importantly, these phenotypes could not have been uncovered through previously published tetraploid complementation experiments [20], which cannot assess the effect of *Med12* loss on extraembryonic lineages. Ultimately, loss of maternal *Med12* leads to failure to maintain trophoblast pluripotency and appropriately allocate trophoblast lineages, disrupting placental architecture and contributing to embryonic mortality.

In our efforts to specifically interrogate the function of maternal *Med12*, we found that embryos from *Zp3Cre;Med12^fl/fl^* dams had persistently low levels of *Med12*, even in the presence of a functional paternal allele. We hypothesized that the rodent-specific phenomenon of programmed paternal XCI [25] compromises embryonic *Med12* expression in embryos lacking maternal *Med12*, effectively generating a maternal-zygotic double knockout. Using a knock-in approach, we showed that expression of an autosomal *Med12* transgene rescues embryonic development from *Med12*-deficient oocytes. This finding indicates that loss of *Med12* in the oocyte does not produce irreversible transcriptomic or epigenetic effects that cannot be overcome by embryonic expression, and that the phenotypes we observed in embryos derived from *Med12*-null oocytes result from a cumulative lack of both maternal and embryonic *Med12*. More broadly, our findings also highlight the challenges of studying X-linked MEG in rodents, where paternal XCI can confound interpretations.

We conclude that oocyte-derived *Med12* is essential for murine development and that *Med12* safeguards not only embryonic signaling programs [20], but also trophoblast pluripotency, lineage integrity, and placental morphogenesis. Whether *Med12* plays a similarly pivotal role during development in other species, such as humans, warrants further investigation, especially given species-specific differences in XCI [47] and placental biology. These findings expand the growing understanding of MEG that contribute to the success of early implantation and placental development in mammals, underscoring the importance of maternal contributions to transcriptional control in early embryonic development.

## Supplementary Material

ioag066_Supplementary_materials

## Data Availability

The RNA-seq data produced in this study are available through the NCBI GEO repository under accession GSE281910. All other relevant data supporting the key findings of this study are available within the article and its Supplementary Data files or from the corresponding author upon reasonable request.

## Notes

**Grant Support:** This work was funded by NIH grant 5P50HD098580. M.M.H. was supported by NIH grant 5T32HD007263-39.
