# Supplementary material for: Maternal Med12 safeguards trophoblast pluripotency and placental development
Source: Biol Reprod. 2026 Mar 24;114(5):1610–20. doi: 10.1093/biolre/ioag066 (PMC13175988; doi:10.1093/biolre/ioag066)

Supplementary Figure 1.

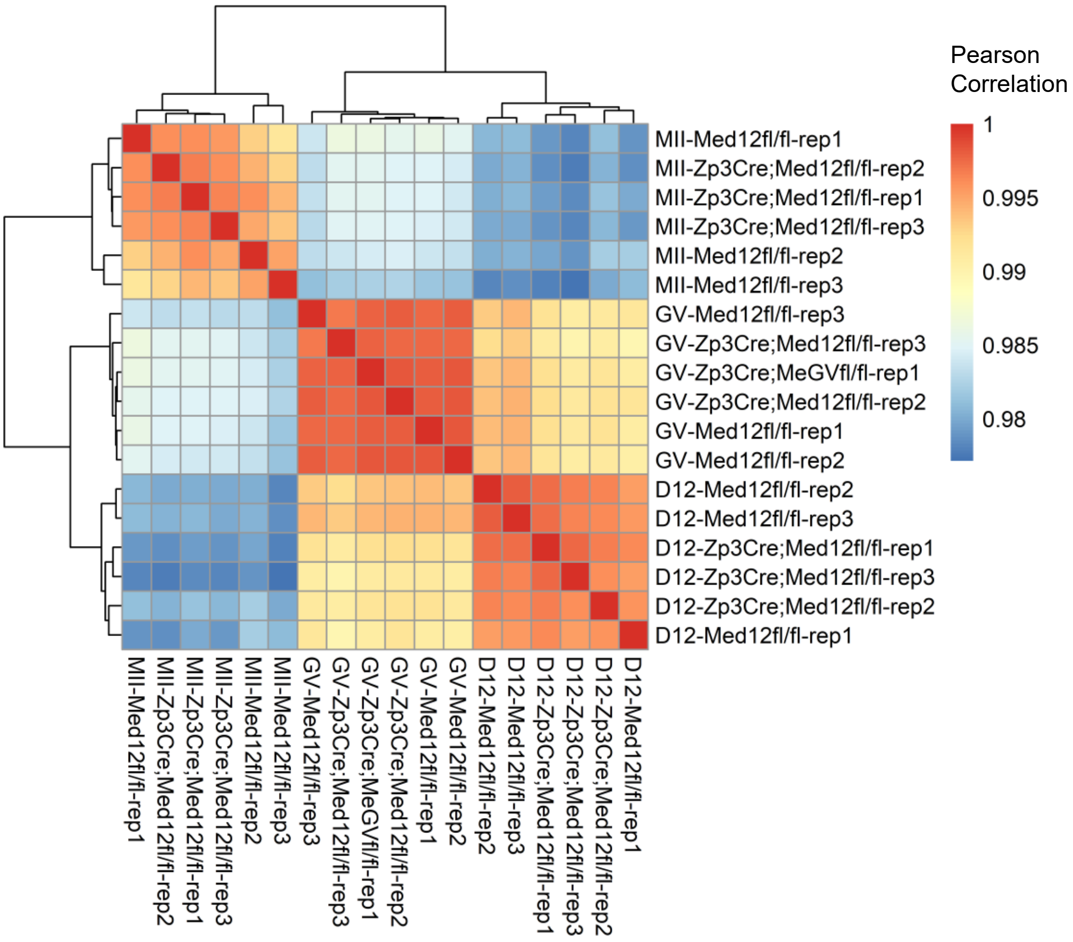

Supplementary Figure 2.

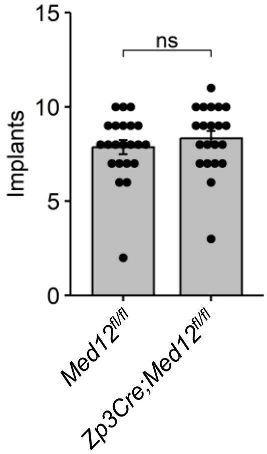

Supplementary Figure 3.

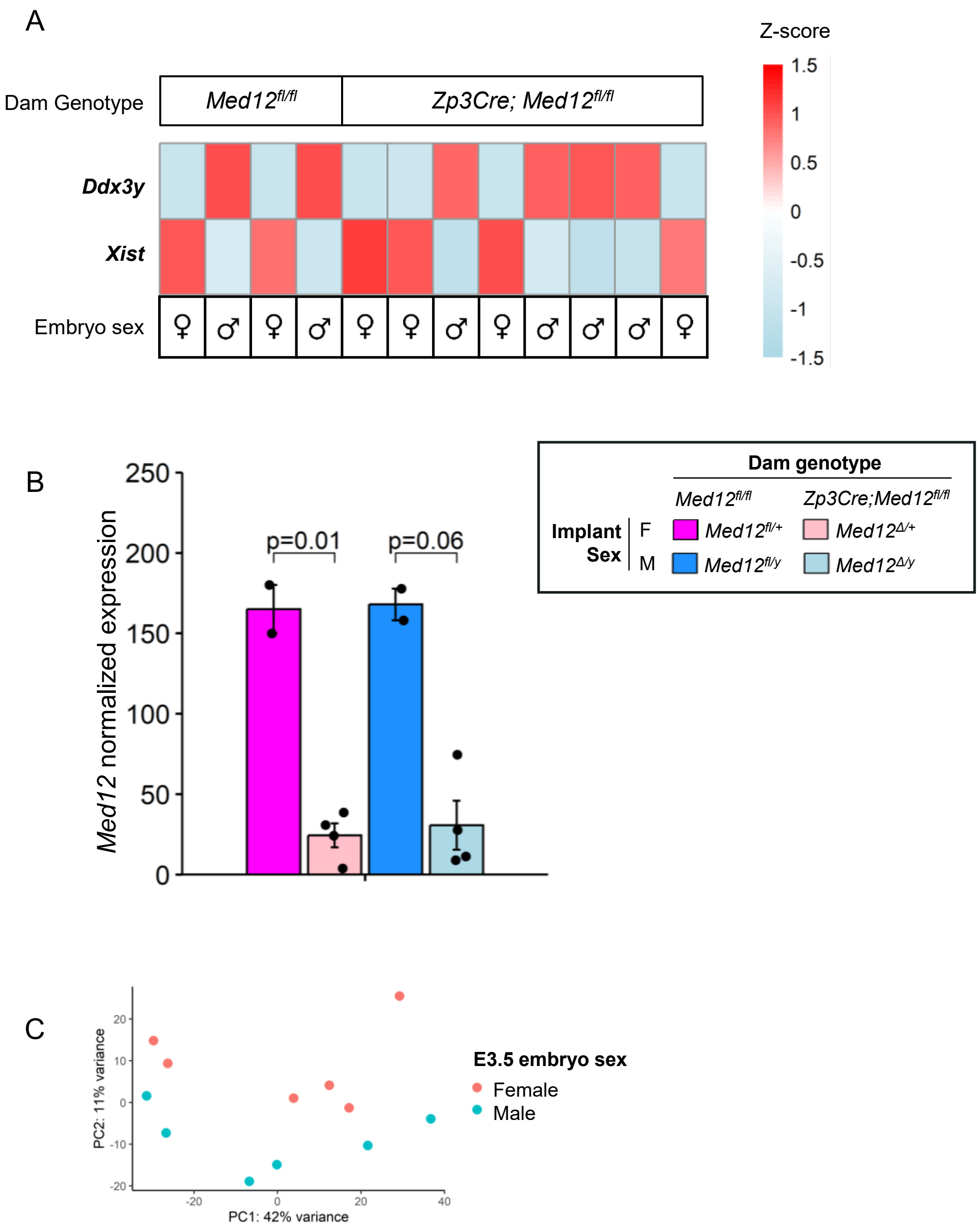

Supplementary Figure 4.

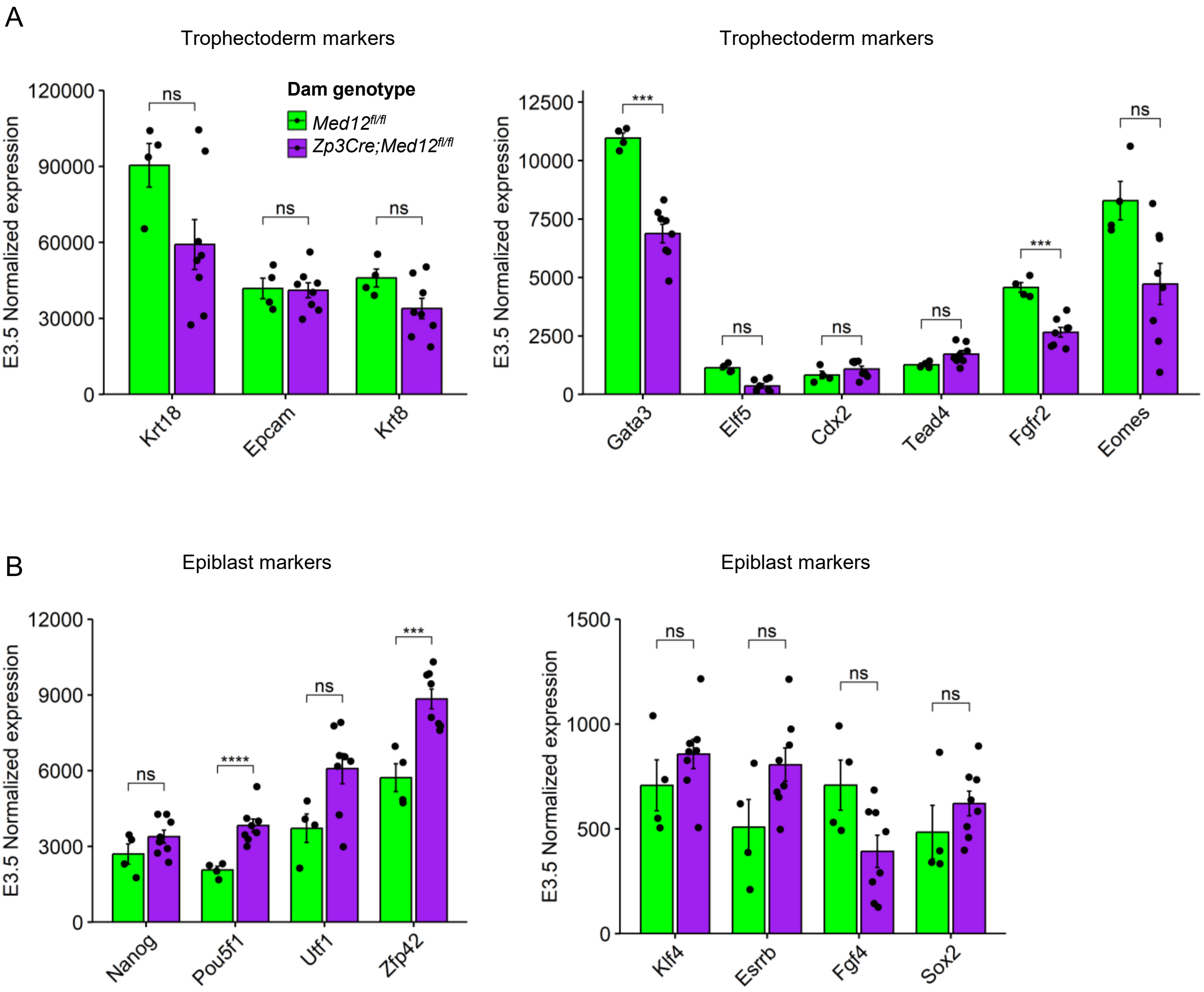

Supplementary Figure 5.

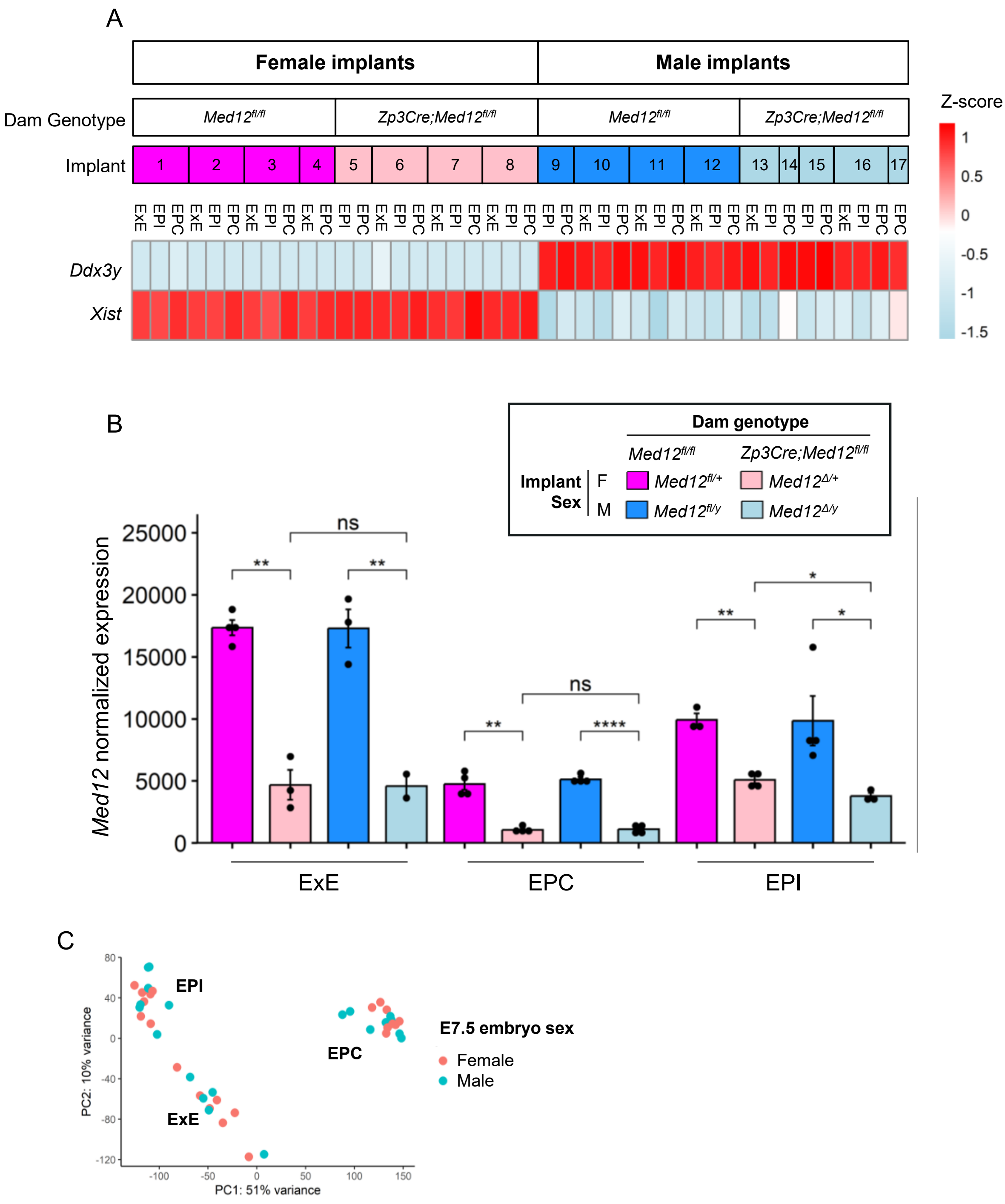

Supplementary Figure 6.

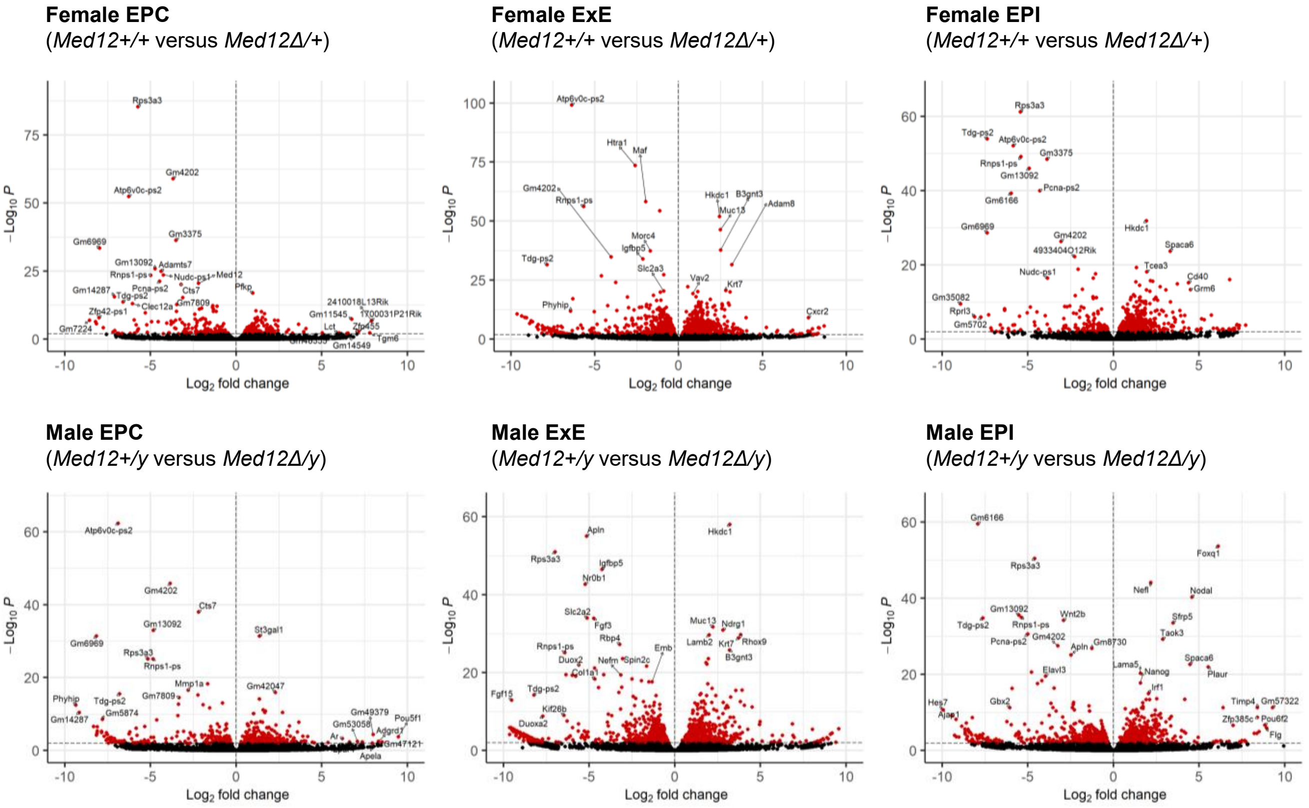

Supplementary Figure 7.

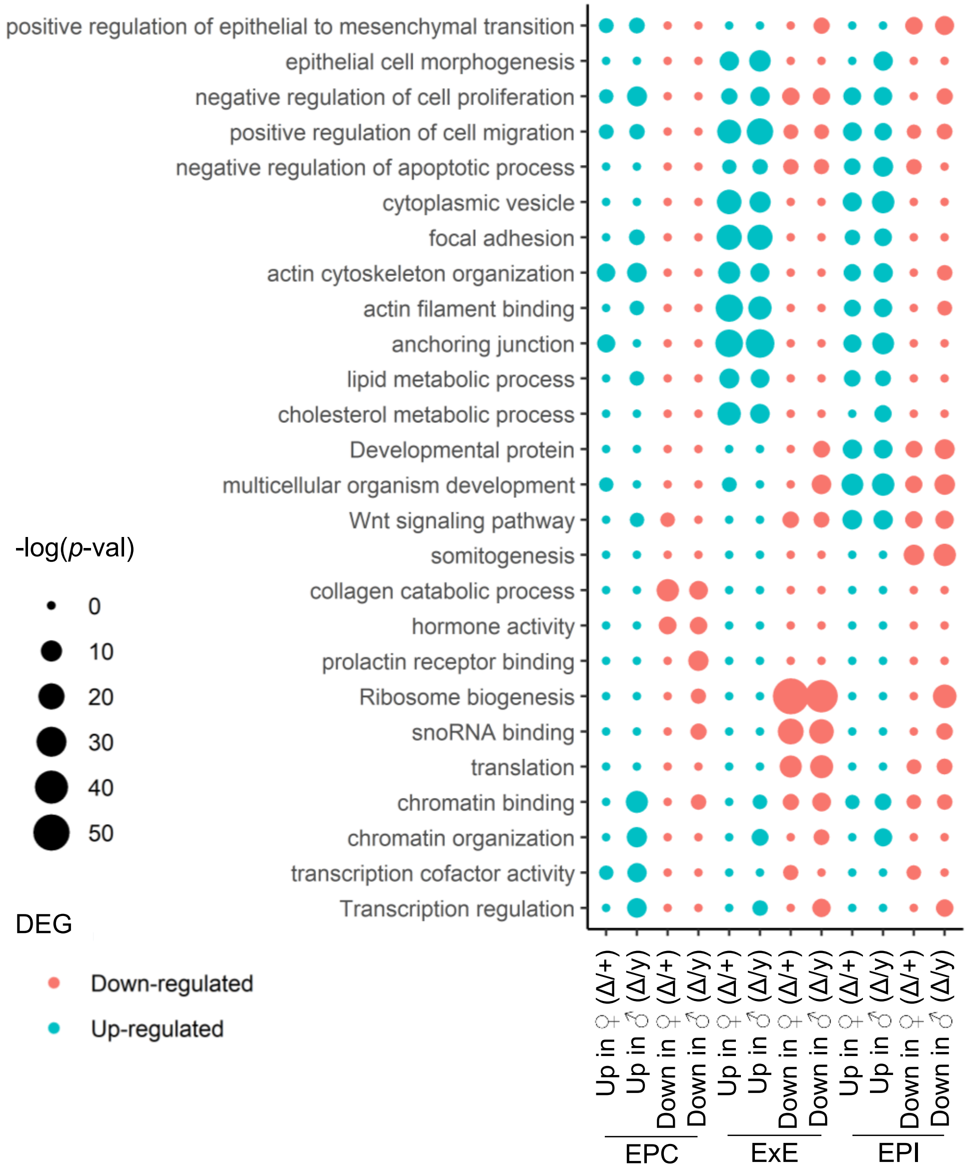

Supplementary Figure 8.

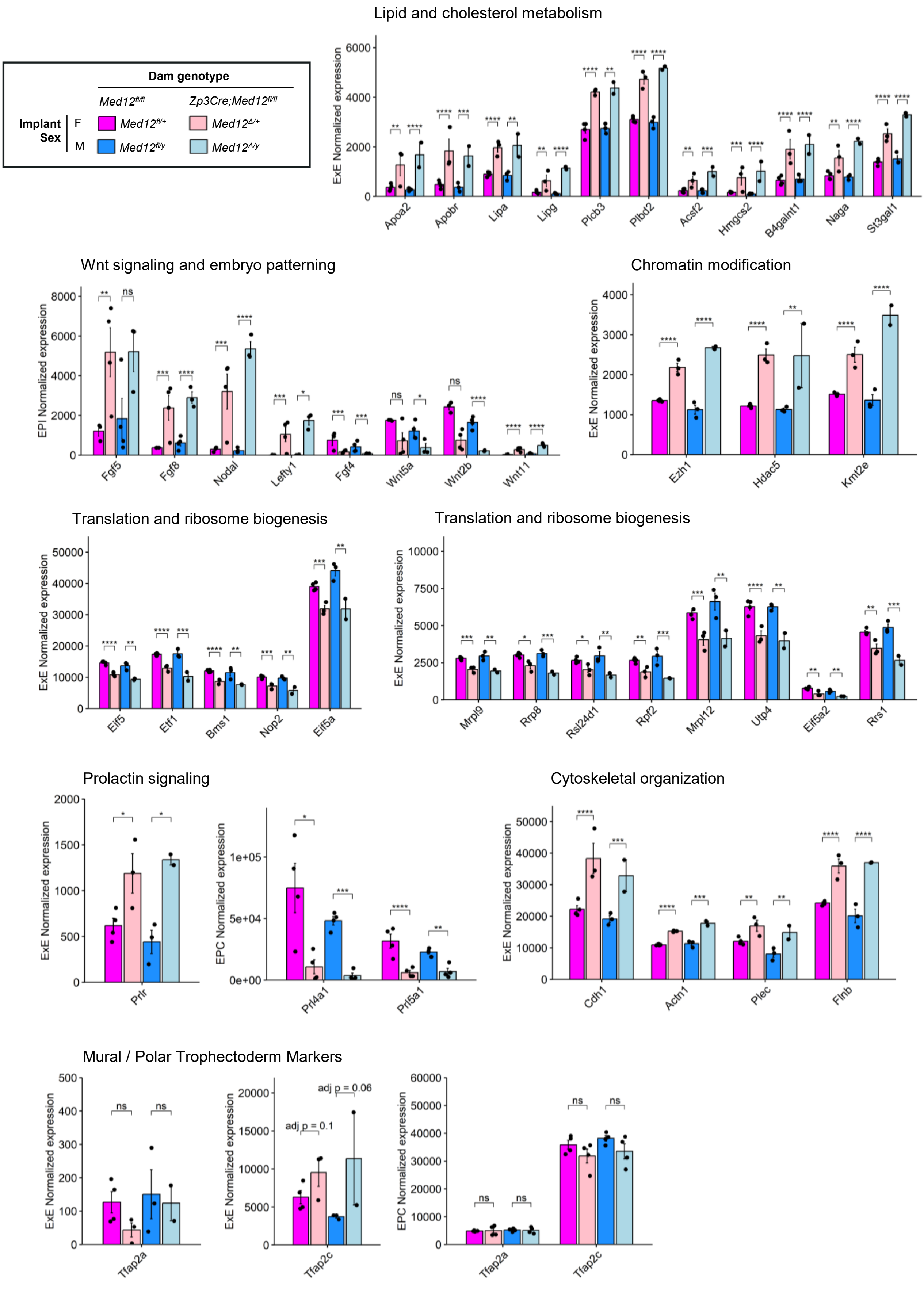

Supplementary Figure 9.

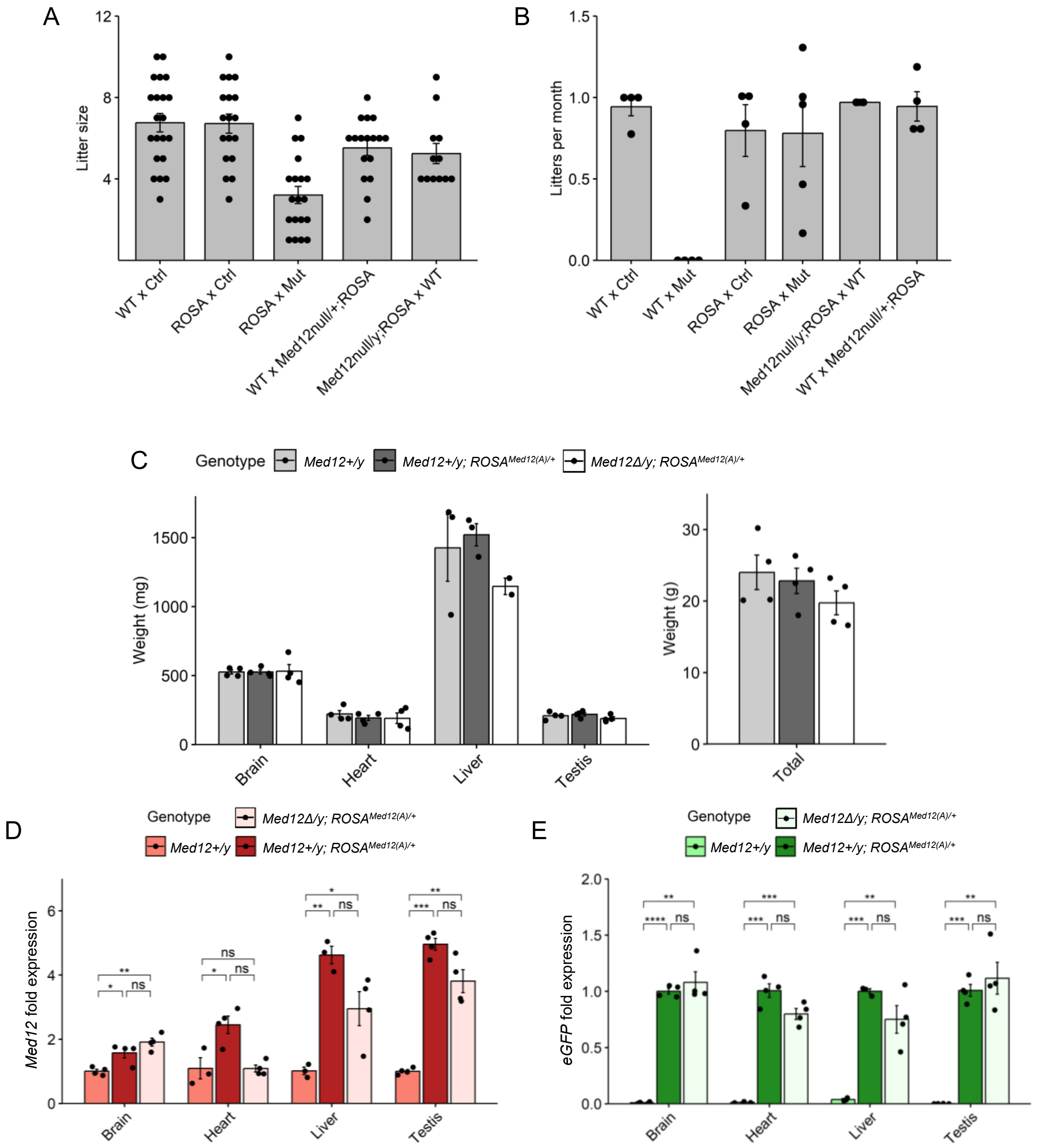

Supplement: ioag066_Supplementary_materials [file ioag066_supplementary_materials.zip › Supplementary_materials_ioag066_Figures.pdf]
