# Supplementary material for: Maternal Med12 safeguards trophoblast pluripotency and placental development
Source: Biol Reprod. 2026 Mar 24;114(5):1610–20. doi: 10.1093/biolre/ioag066 (PMC13175988; doi:10.1093/biolre/ioag066)
Supplement: ioag066_Supplementary_materials [file ioag066_supplementary_materials.zip › Supplementary_materials_ioag066_Tables.docx]

**Supplementary Table 1**. Average embryonic implants identified in control (Med12^fl/fl^) and mutant (Zp3Cre; Med12^fl/fl^) dams at specific developmental timepoints. Two-tailed unpaired student's t-test.

| **Dam Genotype** | **Embryonic stage** | **Dams** | **Mean implants per dam** | **S.E.M.** | ***p*-value** |
| --- | --- | --- | --- | --- | --- |
| Med12fl/fl | E6.5 | 1 | 7 | -- | -- |
| Zp3Cre;Med12fl/fl |  | 2 | 9.5 | 0.50 |  |
| Med12fl/fl | E7.5 | 7 | 8.71 | 0.36 | 0.208 |
| Zp3Cre;Med12fl/fl |  | 8 | 7.88 | 0.88 |  |
| Med12fl/fl | E8.5 | 5 | 7.4 | 0.51 | 0.288 |
| Zp3Cre;Med12fl/fl |  | 2 | 8 | 1.00 |  |
| Med12fl/fl | E9.5 | 6 | 7.67 | 1.17 | 0.243 |
| Zp3Cre;Med12fl/fl |  | 8 | 8.5 | 0.50 |  |
| Med12fl/fl | E12.5 | 2 | 8 | 1.00 | 0.377 |
| Zp3Cre;Med12fl/fl |  | 6 | 7.25 | 1.17 |  |

**Supplementary Table 2**. Primers used for PCR genotyping of mice.

| **Target** | **Primer sequence (5’ to 3’)** | **Amplicon(s)** | **Reference** |
| --- | --- | --- | --- |
| *Med12^flox^* | F: 5’ AGGCACCGAGTACCTGTTCAAGAAT 3'  R: 5' TATCATTCCTGATCCCCATCTTCCT 3' | 313 bp (wild-type)  400 bp (flox) | [20] |
| *Med12^Δ1-7^* (deletion) | F: 5′ GTTTCCGGCAGTAATCGAGAGTTTC 3’  R: 5′ TATCATTCCTGATCCCCATCTTCCT 3′ | 330 bp | [20] |
| *Zp3Cre* | F: 5' GCGGTCTGGCAGTAAAAACTA 3'  R: 5' GTGAAACAGCATTGCTGTCAC 3' | 100 bp | JAX Protocol #22392 |
| *Med12* ROSA knock-in | A: 5' AAAGTCGCTCTGAGTTGTTAT 3'  B: 5' GCGAAGAGTTTCTCCTCAACC 3'  C: 5' GGAGCGGGAGAAATGGATATG 3' | 650 bp (wild-type)  350 bp (knock-in) | [22] |

**Supplementary Table 3**. Primers used for RT-qPCR.

| **Target** | **Primer sequence (5’ to 3’)** |
| --- | --- |
| *Gapdh* | FWD: 5’- CTCCCACTCTTCCACCTTCG -3’  REV: 5’- GCCTCTCTTGCTCAGTGTGG -3’ |
| *Med12* | FWD: 5’- CAGCTGATGAGAAGGGCTCC -3’  REV: 5’- TCGCTCACTTTCACTTCGGG -3’ |
| *eGFP* | FWD: 5’- ACGACGGCAACTACAAGACC -3’  REV: 5’- GGGGTGTTCTGCTGGTAGTG -3’ |
